# Correction: Biochemical characterization of protease activity of Nsp3 from SARS-CoV-2 and its inhibition by nanobodies

**DOI:** 10.1371/journal.pone.0302418

**Published:** 2024-04-16

**Authors:** Lee A. Armstrong, Sven M. Lange, Virginia De Cesare, Stephen P. Matthews, Raja Sekhar Nirujogi, Isobel Cole, Anthony Hope, Fraser Cunningham, Rachel Toth, Rukmini Mukherjee, Denisa Bojkova, Franz Gruber, David Gray, Paul G. Wyatt, Jindrich Cinatl, Ivan Dikic, Paul Davies, Yogesh Kulathu

The third author’s name is spelled incorrectly. The correct name is Virginia De Cesare. The correct citation is: Armstrong LA, Lange SM, De Cesare V, Matthews SP, Nirujogi RS, Cole I, et al. (2021) Biochemical characterization of protease activity of Nsp3 from SARS-CoV-2 and its inhibition by nanobodies. PLoS ONE 16(7): e0253364. https://doi.org/10.1371/journal.pone.0253364.
