# Supplementary figures and images for: Biochemical characterization of protease activity of Nsp3 from SARS-CoV-2 and its inhibition by nanobodies
Source: PLoS One. 2021 Jul 16;16(7):e0253364. doi: 10.1371/journal.pone.0253364 (PMC8284666; doi:10.1371/journal.pone.0253364)

S1 Fig.

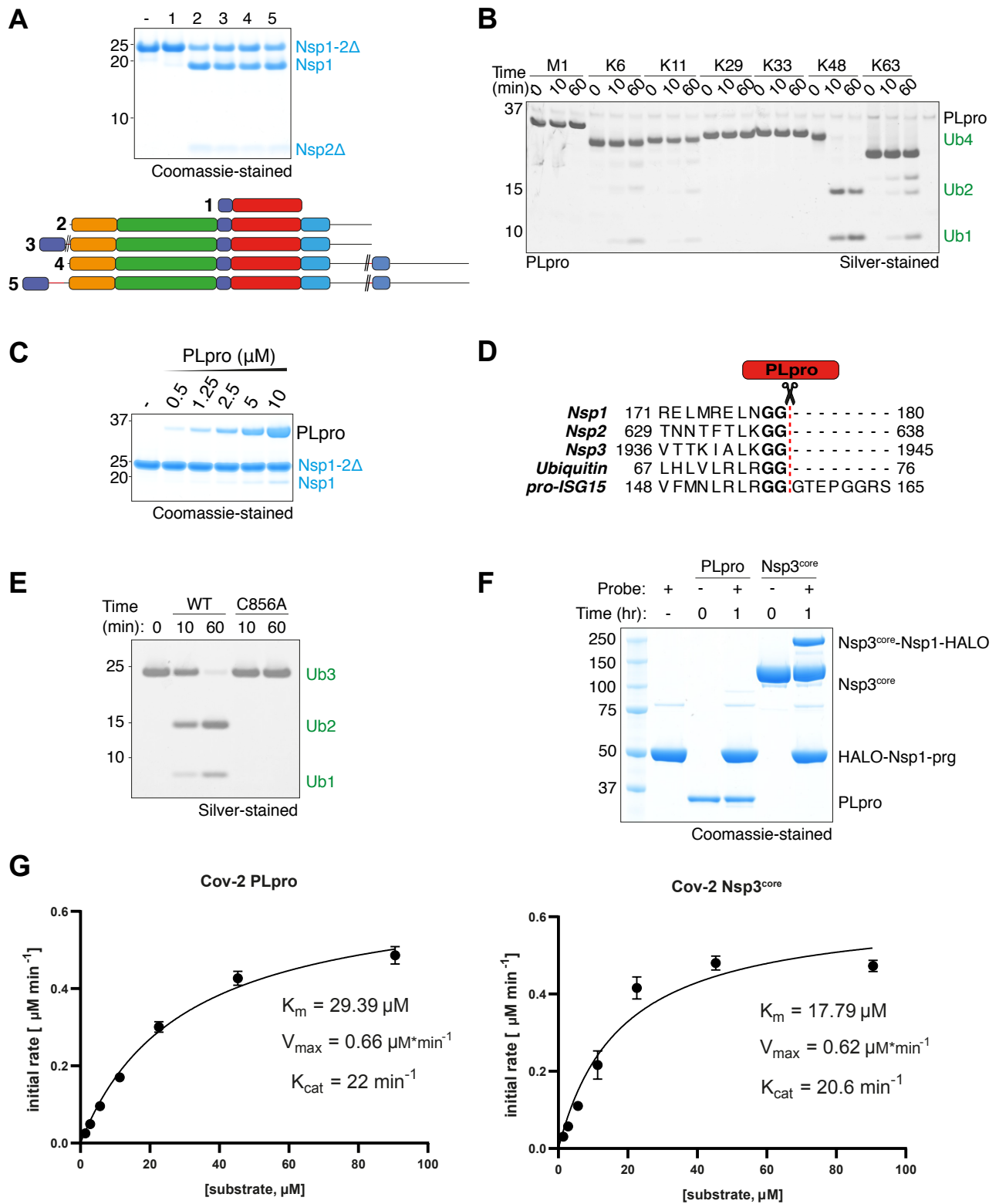

Supplement: S1 Fig — A) Assay showing the cleavage of Nsp1-2Δ by the indicated PLpro (1) and Nsp3 constructs (2–5) (colour-coding as in Fig 1A). B) DUB assay showing the polyubiquitin linkage specificity of PLpro. C) Assay showing the effect of increasing PLpro concentration on cleavage of Nsp1-2Δ visualized by Coomassie staining. D) Sequence alignment of the C-termini of all PLpro substrates highlighting the cleavage site and consensus recognition motif. E) Cleavage assay comparing activity of WT and C856A Nsp3core against K48-linked Ub3. F) Assay monitoring the conversion of PLpro and Nsp3 by a HALO-Nsp1-prg probe. G) Michaelis-Menten kinetics of K48-Ub3 cleavage by PLpro and Nsp3core. (PDF) [file pone.0253364.s001.pdf]

S2 Fig.

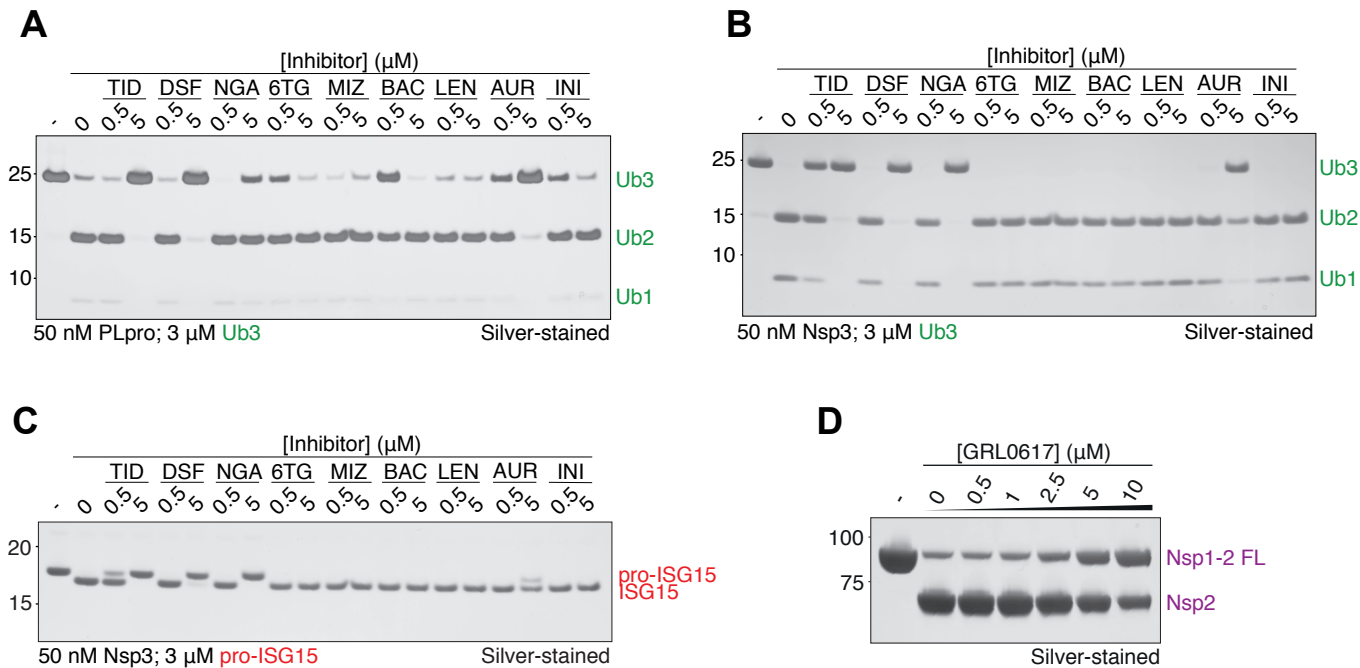

Supplement: S2 Fig — Orthogonal, gel-based assays employing K48 trimer (A, B) and pro-ISG15 (C). Tideglusib (TID), Disulfiram (DSF), Nordihydroguaiaretic acid (NDA), Thioguanine (6TG), Methimazole (MIZ), Bacitracin (BAC), Lenalidomide (LEN), Auranofin (AUR), and Iniparib (INI) were tested at the indicated concentrations against PLpro (A) and NSP3 (B,C). D) Assay testing the ability of GRL0617 to inhibit Nsp1-2 FL cleavage by Nsp3core. Data shown are representative of two independent experiments. (PDF) [file pone.0253364.s002.pdf]

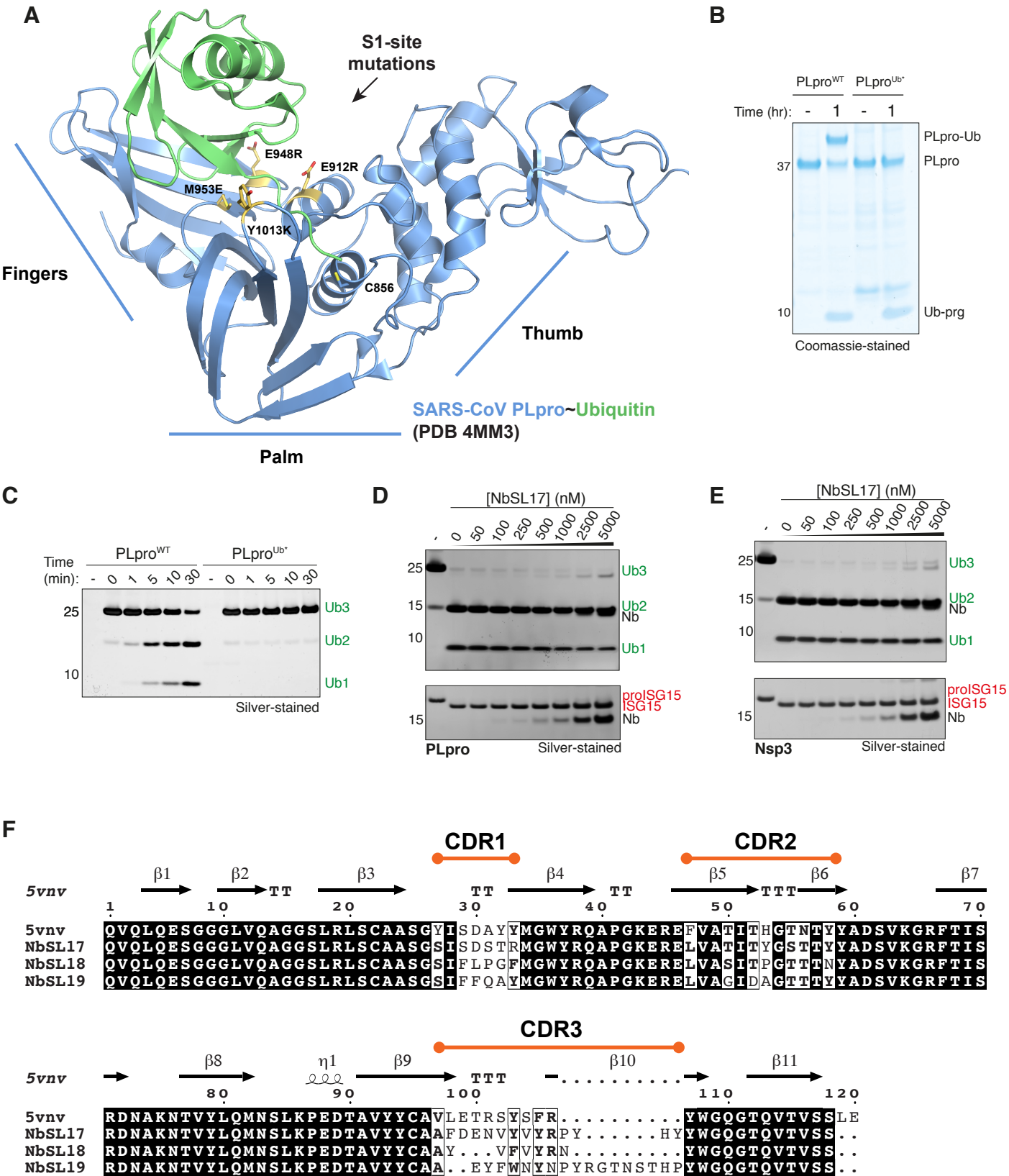

Supplement: S3 Fig — A) Structure of SARS-CoV PLpro (blue) bound to Ubiquitin (green) (PDB 4MM3). Stick models show mutated S1-binding site residues (gold) and catalytic cysteine C856. Labels indicate right-hand architecture of PLpro with Fingers, Palm and Thumb subdomains. B) Coomassie stained gel comparing reactivity of PLpro WT and the S1* mutant with Ub-prg. C) DUB assay of PLpro WT and the S1* mutant with K48-Ub3 as substrate visualized by silver staining. D-E) DUB assays of PLpro (D) and Nsp3core (E) with K48-Ub3 (top) and proISG15 (bottom) in the presence of increasing concentrations of NbSL17. F) Sequence alignment of identified inhibitory nanobodies NbSL17, NbSL18 and NbSL19 with synthetic nanobody Nb.201 (PDB 5VNV). Secondary structure elements and location of complementarity determining regions (CDRs) are indicated above the sequence. (PDF) [file pone.0253364.s003.pdf]
