# Supplementary material for: Biochemical characterization of protease activity of Nsp3 from SARS-CoV-2 and its inhibition by nanobodies
Source: PLoS One. 2021 Jul 16;16(7):e0253364. doi: 10.1371/journal.pone.0253364 (PMC8284666; doi:10.1371/journal.pone.0253364)
Supplement: S1 Table — (PDF) [file pone.0253364.s004.pdf]

S1 Table: List of cDNA constructs

Protein	Expressed protein	Tag cleaved	Vector type	Plasmid	DU number
PLpro	His_6-GST-Nsp3 (743-1072)	Yes	Bacterial	pETDUET	67811
PLPro	His_6-GST-AVI-Nsp3 (743-1072)	Yes (His_6/GST)	Bacterial	pETDUET	67765
PLpro (E912R E948R M953E Y1013K)	His_6-GST-AVI-PLpro (E912R E948R M953E Y1013K)	Yes (His_6/GST)	Bacterial	pETDUET	67766
Nsp3 (179-1329)	Twinstrep-Nsp3 (179-1329)-His_6	No	Bacterial	pET15D	67831
Nsp3 (179-1329) C856A	Twinstrep-Nsp3 (179-1329)-His_6C856A	No	Bacterial	pET15D	67898
Nsp3 (1-109-linker-179-1329)	Twinstrep-Nsp3 (1-109-linker-179-1329)-His_6	No	Bacterial	pET15D	67844
Nsp3 (179-1329-linker-1584-1944)	Twinstrep-(179-1329-linker-1584-1944)-His_6	No	Bacterial	pET15D	67847
Nsp3 (1-1329-Linker-1584-1944)	Twinstrep-Nsp3 (1-1329-linker-1584-1944)-His_6	No	Bacterial	pET15D	67881
Nsp1-2 (1-19)	Nsp1-Nsp2 (1-19)-His_8	No	Bacterial	pET28A	67799
HALO-$Nsp1^{prg}$(1-179)	HALO-Nsp1 (1-179)-intein-CBD	No	Bacterial	pTXB1	67780
Nsp1-2 FL	His_8-GST-Nsp1-2	Yes	Bacterial	pET15D	70079
Ubiquitin	Untagged ubiquitin	-	Bacterial	pET24	20027
Pro-ISG15	GST-proISG15	Yes	Bacterial	pGEX6P1	8371
Empty vector	mCherry	-	Mammalian	pcDNA5 FRT/TO	41458
Nsp3 (1-1329-linker-1584-1944)	Twinstrep-Nsp3 (1-1329-linker-1584-1944)-His_6	No	Mammalian	pcDNA5 FRT/TO	67879
PLpro	Twinstrep-Nsp3 (743-1072)	No	Mammalian	pcDNA5 FRT/TO	67916
