# Supplementary material for: Biochemical characterization of protease activity of Nsp3 from SARS-CoV-2 and its inhibition by nanobodies
Source: PLoS One. 2021 Jul 16;16(7):e0253364. doi: 10.1371/journal.pone.0253364 (PMC8284666; doi:10.1371/journal.pone.0253364)
Supplement: S2 Table — (PDF) [file pone.0253364.s005.pdf]

S2 Table: Details of reagents

Reagent or Resource	Source	Identifier
BL21-CodonPlus (DE3)-RIL Competent Cells	Agilent	Cat# 230245
Propargylamine	Sigma Aldrich	Cat# P50900-5G
MESNa	Sigma Aldrich	M1511-25G
Desthiobiotin	IBA Life Sciences	Cat# 2-1000-002
Strep-Tactin Sepharose	IBA Life Sciences	Cat# 2-1201-010
His60 Ni Superflow Resin	TaKaRa	Cat# 635659
Recombinant Human IFN-α1	Cell Signalling Technologies	Cat# 8927SC
Lipofectamine 2000	ThermoFisher Scientific	Cat# 11668019
