# Supplementary material for: Biochemical characterization of protease activity of Nsp3 from SARS-CoV-2 and its inhibition by nanobodies
Source: PLoS One. 2021 Jul 16;16(7):e0253364. doi: 10.1371/journal.pone.0253364 (PMC8284666; doi:10.1371/journal.pone.0253364)
Supplement: Attachment — Submitted filename: 112875_1_rebuttal_2009234_qpg9sc.docx [file pone.0253364.s006.docx]

------------------------------------------------------------------------------ 
Reviewer #1 (Evidence, reproducibility and clarity (Required)): 

The Nsp3 protein of SARS-CoV-2 contains a papain-like protease domain, PLpro, that is involved in the cleavage of (1) the viral polyprotein as well as (2) ubiquitin and (3) ISG15 from host proteins involved in the antiviral response. Here, the authors show that additional domains of the SARS-CoV-2 Nsp3 may play a role in these cleavage activities, as a near full-length Nsp3 cleaves all three substrates more efficiently than the PLpro domain alone. Despite differences in cleavage activity, an interactome analysis of the Nsp3 protein (lacking the transmembrane domain) and the PLpro domain alone found only one unique interacting protein of Nsp3. The authors then screened a library of FDA-approved inhibitors for their ability to restrict PLpro ubiquitin-cleavage activity and found that five inhibitors could reduce both PLpro and Nsp3 enzymatic activity. However, these inhibitors had high off-target inhibition of host deubiquitinating enzymes and did not restrict SARS-Cov-2 replication. The authors then identify a S1 site-specific, soluble nanobody that inhibits PLpro- and Nsp3-mediated cleavage of ubiquitin and ISG15. 

While the authors provide interesting insights into the cleavage activities of Nsp3 and its PLpro domain, the paper is lacking evidence regarding the physiological significance of these cleavage activities on both the antiviral immune response and virus replication. Many data presented are, unfortunately, negative data. Thus, the study as it stands is expected to have only a limited impact on the field. 
We thank the reviewer for their comments. We disagree that many data presented in this manuscript are negative data. The only piece of negative data is the one where the inhibitors which show good inhibition in biochemical assays do not show any effects in restricting viral replication. We still consider it important information to share with the wider research community to encourage vigilance in ongoing drug screens to test in vitro lead compounds in viral replication assays as early as possible to prevent misallocation time and resources. This manuscript provides several pieces of new information that advances our understanding of Nsp3, such as the identification of cleavage activity observed with Nsp3 that is not present in the minimal PLpro domain, the use of more physiological substrates, the engineering of nanobodies that bind to and inhibit Nsp3 activity.
**Major/General Comments:** 

1. The NbSL18 nanobody should be tested for its ability to enhance antiviral IFN/ISG induction and to restrict SARS-CoV2 replication. While the inhibition of PLpro and Nsp3 enzymatic activity by the nanobody is striking, the lack of physiological evidence limits the impact of the findings.

Demonstrating physiological significance goes beyond the scope of this manuscript. If required, we are happy to change the title to “Biochemical characterization….” to better reflect the data presented in the manuscript. 
2. Several edits should be made to the text to make the scientific logic more comprehensive. See figure-specific points in the next section and major points below: 
a. In the introduction, please elaborate on how ubiquitination and ISGylation control the antiviral response. Please provide examples of antiviral proteins that are post-translationally modified in this way. 
We will include additional information on roles of ubiquitylation and ISGylation in the antiviral response.
b.Figures are referenced out of order in the text (e.g., Fig 1B is mentioned before Fig 1A; Fig 3D is mentioned before Fig 3C). 
We thank the reviewer for pointing this out and will change the order of the figures to fit the flow of the narrative in the results section
c.The Nsp3 spanning residues 179-1329 should not be "henceforth referred to as "Nsp3" as this suggests it is the wildtype full-length protein. Please designate this Nsp3 truncation more clearly. 
We will refer to the truncated Nsp3 as Nsp3^core^ to avoid any confusion
**Specific points:** 

Figure 1: 
•Fig 1C: It is unclear from the text why all four Nsp3 truncations were made/shown but only one was chosen for further characterization.

We made 4 Nsp3 truncations to test solubility and expression. The four truncations were assayed for activity and showed similar activity. We chose the truncation that expressed best and was also the one that we were using for crystallization trials.

•Please include in the text a hypothesis for which additional domains contained in the Nsp3 179-1329 mutant, but not in PLpro alone, may contribute to the observed Nsp1-2 cleavage.

Since we do not have any idea of which domains could be contributing to the observed Nsp1-2 cleavage, we were not comfortable speculating.

Figure 2: 
• Please include a blot showing equal expression of the extended Nsp3 and PLpro analyzed by quantitative mass spectrometry or an explanation in the text to ensure expression differences do not contribute to the difference in interactor enrichment.
We will include a gel showing expression levels of Nsp2 and PLpro in the input samples
• Please more clearly describe the data in the figure legend to emphasize that the significant interactors are all overlapping interactors with slightly different enrichments between the two Nsp3 truncations. FXR1 can be colored/labeled differently to indicate it was the only unique hit in the Nsp3 pull-down.

We thank the reviewer for the suggestion and we will highlight this accordingly.

• Authors may want to highlight in the text that the IFITs are previously-characterized substrates for ISG15. 
We thank the reviewer for pointing this out and will include this information.

Figure 3: 
• This figure and the related text should focus on inhibitors that restrict SARS-CoV-2 replication, such as GRL0617. Negative data should be mentioned briefly in the text and moved to the supplementary information. 
These experiments demonstrate how the MALDI TOF assay can be used to screen for inhibitors. Further, it makes an important point that identification of a good inhibitor in vitro does not necessarily imply restriction of viral replication. The information presented on the compounds could serve as the starting point to improve potency of the identified compounds. Lastly, it shows that none of these FDA approved compounds can be immediately repurposed and shows how these compounds can inhibit several human deubiquitinases, which we believe is important information to publish.

**Minor Points:** 
• Please provide a brief description of "nanobody" in the text (Figure 4).

Yes, we will include a description of nanobodies
• In the text, please change "right" to "native" in the explanation for why the different Nsp3 proteins did not have many unique interactors (Figure 2). 
OK

Reviewer #2 (Evidence, reproducibility and clarity (Required)): 

**Summary:** 

In this manuscript the authors present biochemical and cell base assay data on various aspects of the papain-like protease (PLpro) from SARS-CoV-2, the causative agent of COVID-19. This includes performing various assays based on the cleavage of poly-ubiquitin (Ub), interferon stimulated gene product 15 (ISG15), and viral polypeptide constructs of SARS-CoV-2. The latter was particular novel and could add to the knowledge of PLpro from SARS-CoV-2. They also perform a small high-throughput screening campaign identifying several small molecule inhibitors of SARS-CoV-2 PLpro. Finding that these are not specific and do not possess antiviral activity, the authors seek to identify a nanobody that can inhibit PLpro. From a grammar and spelling prospective, the article is well written. However, the are several major issues with the content of the manuscript and the portrayal of that content. Without correction, these issues may inadvertently mislead EMBO's audience. 

**Major comments:** 

1. Lack of referencing and portrayal of several finding as novel for PLpro specificity data. The manuscript appears to ignore several widely cited publications on SARS-CoV-2 and does not appear to put their findings in the context of what has already been known about this enzyme. The biochemical activity of PLpro for ubiquitin and ISG15 as well as specific polypeptide has published last May and October. The latter was in Rut et al. Oct 2020 Sciences Advances, where the authors detail the polypeptide site preference of PLpro. The former Freitas et al. ACS May 2020 inf dis details the biochemical specificity of PLpro for its polymeric Ub and ISG15 substrates. Hence, why Shin et al. July 2020 Nature references Freitas et al. when presenting their results. An additional example of not putting the context of their findings into what is already known in the field is the authors proISG15 assay. This type of assay was already performed by Freitas et al.. for SARS-CoV-2 in more depth and for PLpros in other SARS-CoV-1 publications. The authors do not recognize or compare their findings to what is already known regarding this type of activity. Overall, the authors present their findings for Ub and IS15 as well as certain parts of polypeptide cleavage specificity as novel, when they are confirmatory in nature at best. To address this issue, the authors should place their findings in the context of those found by Rut et al., Freitas et al., and even to a greater extent than they have with Shin et al.. Their assay regarding near full-length polypeptide cleavage of PLpro as well as the interactome is the real novelty in these sections and should be highlighted.

We apologize for the oversight of not citing these additional papers and will include them in the revised manuscript to place our findings in context of these papers. We will also highlight the novel findings of our manuscript better.
2. HTS screen and potential of inhibitors identified. The authors are successful in identifying several compounds from their screen with inhibitor PLpro activity. However, these already have specificity for a myriad human enzyme targets. Inhibition of PLpros though compounds containing warheads for covalent modification has been widely known, so has their inability for use as viable therapeutics for antiviral activity. For instance, the authors do correctly note that 6-thioguanine was already observed to inhibitor of PLpros. However, this is a chemotherapeutic with significant side effects. Side effects that are only accepted because it is a cancer therapeutic, not an antiviral. The authors should seek to better clarify the practicality of these agents. The authors should also seek to identify their mode of action (i.e. confirm that they are covalent modifies, which they likely are). This would be straightforward to resolve with enzymatic assays. 
Where inhibitors could be covalent modifiers, we have mentioned this as the possible mode of action. As these compounds do not show any effects in cells to restrict viral replication, we do not see the benefit of performing these additional experiments.
The authors should also clarify where GRL-0617 originated and their motivation for using it as a comparison. This molecule and others originated from Ratia et al. PNA 2008 were first shown to inhibit PLpro from SARS-CoV-2 in Freitas et al. as well as later articles by Shin et al. and Gao et al. Acta Pharm Sin B Sep 2020. 
We will cite these papers.
3. Use of nanobodies. The authors are successful in developing nanobodies that inhibit PLpro activity. In addition to the interactome, this is the other real highlight of the manuscript. This along with the interatome should be the central points of the manuscript as they are clearly novel and would added to what is known about PLpro from SARS-CoV-2. However, there is very little discussion related to the implications of these discoveries. PLpro and the rest of the nsp3 is anchored within double membrane vesicles inside of the cells where SARS-CoV-2 is replicating. Is there a way that the authors see these nanobodies either accessing the cell, or being manufactured in a way that they could interact with PLpro? The authors do mention use of the nanobodies as a tool. If so, how would they use a nanobody that blocks PLpro function?

We thank the reviewer for the suggestion and will expand on how these nanobodies can be used to block PLpro function in cells.
**Minor comments:** 

4. ITC methods and results. The ITC results the authors seek to publish related to PLpro and its ISG15 and Ub substrates is very problematic given what is already known about PLpros from SARS-CoV-2 and others. As demonstrated by Shin et al. and Freitas et al. as well as others, PLpros from SARS-CoV-2 and SARS-CoV-1 can be product inhibited (i.e. K48 di-Ub out competes other cleavable substrates). Their own biochemical data also highlights this phenomenon with a buildup of K48 entities in their assays. This along with Shin et al solving a noncovalent complex of SARS-CoV-2 PLpro with an ISG15, which would require relatively high affinity, further highlights the concern about reproducibility, the authors methodology, or veracity in conducting the ITC experiments. 
Looking at the ITC methods listed in the manuscript there are a number of concerns. First, the authors are very vague and lack do not include numerous important details. These include, protein concentrations, injection regimens, etc. The nanobody binds in the nanomolar range where as Ub and ISG15 likely bind in the micromolar range. The amount of heat given off by an Ab - ligand binding event could be very different than that of a substrate binding event. Also, there is zinc does not appear to be included in their method. Zinc has been shown to be important for PLpro binding of Ub and ISG15 substrates because PLpro utilizes a zinc finger in the substrate 1 site. This may be another reason among the various ones why the authors see straightforward Ab binding but appear not possible to measure Ub, or ISG15 binding. The authors could be suggesting that Ub and ISG15 doesn't bind to PLpro after nanobodies bind, but that isn't clear either. Greater clarification of their methods and whether they accounted for all the possibilities of why their findings seem to contradict numerous other reports would be needed. This may include the need to reinvestigating, or remove, the Ub and ISG15 related ITC results. 
We respectfully disagree with the reviewer on this. When the proteins were expressed in *E.coli*, we added 50 µM zinc chloride to the culture. Subsequently, we purified the proteins without adding any EDTA that may chelate away zinc. We have previously worked with ubiquitin binding zinc fingers and DUBs containing zinc fingers and measured their affinity for ubiquitin using ITC and we do not include zinc in these buffers (for example see Kristariyanto et al Mol Cell 2015, Kwasna et al Mol Cell 2018). However, to satisfy the reviewers concerns, we will repeat the binding measurements by including zinc in the buffers and also use an additional method such as microscale thermophoresis to confirm the ITC results. We will also include the missing details in the methods section for the ITC experiments.
