# Supplementary material for: Biochemical characterization of protease activity of Nsp3 from SARS-CoV-2 and its inhibition by nanobodies
Source: PLoS One. 2021 Jul 16;16(7):e0253364. doi: 10.1371/journal.pone.0253364 (PMC8284666; doi:10.1371/journal.pone.0253364)
Supplement: Attachment — Submitted filename: PLOS ONE response to Reviewer Comments.docx [file pone.0253364.s007.docx]

**Response to Reviewer’s comments:**

We would like to thank the reviewers for their interest in our work and for their constructive comments and suggestions, which have allowed us to strengthen our findings and improve the manuscript. We enclose a revised manuscript that addresses the issues that they raised and a point-by-point response addressing each of their comments is detailed below.

Reviewer #1: The manuscript by Armstrong et al., provides an in-depth biochemical characterization of the papain-like protease activity (PLpro) within the Nsp3 non-structural protein of SARS CoV-2 towards different substrates (poly-ubiquitin, ISG15 and viral proteins). The authors found that additional domains within Nsp3 are required for the full protease activity against all 3 tested substrates. Despite the observed differences in protease activities, proteomic analysis for the interacting proteins for PLpro and Nsp3 showed a rather similar interacting proteome. They then applied a mass-spectrometry based approach to identify inhibitors for the PLpro activity, using a library of almost 2000 FDA approved compounds. They managed to select 5 inhibitors with relative specificity towards PLpro, which however did not show any anti-viral effects. Finally, the authors using a yeast-based nanobody library they selected nanobodies that bind to the substrate binding site of PLpro and effectively inhibit PLpro activity.

In general, this is a very well performed study and the quality of presented data is excellent. The in-depth biochemical analysis revealed new information on the protease activity within Nsp3, indicating distinct mechanisms for the proteolysis of different substrates. Despite the unfortunate results on the use of the developed inhibitors, I think the used pipeline provides a useful platform for the community for future screens for PLpro inhibitors. The development of nanobodies is clearly a significant advance in the field and a proof of principle for the use of the yeast nanobody libraries to select potent inhibitors.

We thank the reviewer for the positive comments and feedback.

Based on the comments of the Reviews Common platform, I feel the major issue is to accurately describe the literature on the activity of PLpro and potentially discuss the prospect of the use of the developed nanobodies in vivo. The authors have also provided a satisfactory response on how they will also address any raised technical issues/concerns.

We have now added additional references and described the literature on the activity of PLpro and the inhibitor GRL-0617. We have also added a discussion on how the nanobodies could be used to get a better understanding of the roles of Nsp3 in viral infection. We have also addressed the comments of the reviewers from Reviews Common as described in the previous response to reviewers

Reviewer #2:

Comments:
The ongoing COVID-19 pandemic caused by SARS-CoV-2 has spread worldwide. Effective therapeutic reagents are urgently needed. The Nsp3 with a papain-like protease PLpro domain cleaves the viral protein and polyubiquitin and ISG15 from host cells and, therefore considered as a promising antiviral target. The authors systematically characterized the enzymatic activity of Nsp3 and PLpro of SARS-CoV-2, and found that Nsp3 is more active compared to its PLpro domain, especially for the Nsp1/2 cleavage. Five compounds have been found to inhibit PLpro from a screening of 1971 approved clinical compounds. Further, the authors developed nanobodies that target the active site of PLpro and inhibited the activity of this enzyme. This work provided important information of Nsp3/PLpro catalytic specificity, and demonstrated a promising method for developing anti-PLpro nanobodies.
We thank the reviewer for their positive assessment of our work.

Major suggestions:

1. Can the authors discuss a bit more on possible reasons that why Nsp3 has more active protease activity compared to PLpro?

It is intriguing that the longer versions of Nsp3 are more active than PLpro. Based on sequence analysis, we are unable to identify the reason. In the absence of a crystal structure or other convincing evidence, it is difficult to speculate and so we have left it the way it is.

2. It is interesting that PLpro is unable to cleave Nsp1-Nsp2, but the full-length Nsp3 can. Can the authors discuss a bit more about the possible reasons?

We have now included a brief discussion where we speculate that one possibility could be that other domains such as the SUD domain could be mediating interactions with Nsp1 and Nsp2, which may explain why longer versions of Nsp3 can cleave but minimal PLpro cannot.

3. The engineered competitive nanobodies, especially for NbSL18, showed inhibition on PLpro enzyme activity. It would be interesting to show its antiviral activity in future studies.

We thank the reviewer for the suggestion. This is very much our intention. As these nanobodies only show 50% inhibition at ~500nM, we are presently performing affinity maturation to improve the inhibition by these nanobodies. We intend to share these improved nanobodies with virologists to test for antiviral activity.

Minor suggestions:

1. Introduction, “While several coronaviruses express two PLpro enzymes, SARS-CoV-2 only expresses one PLpro enzyme (Barretto et al, 2005).”
Can the authors add another reference after 2020 to show SARS-CoV-2 only has one PLpro?

We thank the reviewer for the suggestion and have added additional references.

2. Introduction, “PLproCoV-2 shows many similarities to the PLpro encoded by SARS, PLproSARS.”
Please change “by SARS” to “by SARS-CoV”, as SARS is the name of the disease and SARS-CoV is the name of virus.

We have changed this now.

3. “Excitingly, our data reveals that NbSL18 can indeed inhibit Nsp1-2 FL cleavage by Nsp3 suggesting that the viral peptide also occupies the same S1 site as ubiquitin (Fig 4H).”
Can the authors add labels to Fig 4H?

We have added this now.

Reviewer #3: The manuscript “Characterization of protease activity of Nsp3 from SARS-CoV-2 and its inhibition by nanobodies” by Armstrong et al. compares the enzymatic activity of PLpro vs. Nsp3 towards three substrates; compares the protein interactome of non-membrane-anchored PLpro vs. Nsp3; identifies pharmacological inhibitors of PLpro in vitro; and identifies and characterizes inhibitory nanobodies in vitro.

The amount of data and methodological range is impressive and the data as such seem technically sound. Some of the findings turned out as dead ends, e.g. the inhibitor study which did not yield any candidate that works in cells. However, I agree with the authors’ response to one of the previous reviewers that it is important to report these data.
While there are some interesting findings on which one could have built on (especially the interactome data and the new nanobodies as research tools), the authors decided to limit themselves to discuss existing literature, formulate hypotheses or suggest experiments that could be done. Hence, the manuscript as presented is rather broad and leaves the reader with some loose ends. Still, the data and new reagents might be useful for researchers in the field.

We thank the reviewer for their comments and feedback.

Major comments:

- The promised changes in the answers to the previous reviewers’ comments should be implemented. Particularly fair referencing of existing studies and putting the results in the right context is absolutely essential.

We agree and have addressed these concerns raised by the previous reviewers and cited published studies and rewritten our results section in parts.

- Page 18/figure 4G: How were the values for ~50% inhibition determined/quantified? By visual inspection of the gel bands for Ub3/2/1, it seems more like 2500nM to me.

The value for ~50% inhibition was estimated by eye based on the amount of Ub3 cleaved and amount of Ub1 formed. We think its between 500-1000nM

- Page 19: The statement “… thus explaining…” suggests certainty. As other possible explanations are possible, this statement should be toned down.

We thank the reviewer for the suggestion and have changed this to “potentially explaining”

- For the most part it is not clear how often (or whether) experiments were repeated and what the error bars mean (where applicable).

We apologise for this omission and have now included this information in all figure legends.

Minor comments:

- Emotional or scientifically uninformative phrasing should be avoided and/or replaced by (semi-) quantitative or specific terms where appropriate (“…very active…”, “To our disappointment…”, “Excitingly…”, “suitable buffer” etc.)

We have removed or replaced such phrasing as suggested.

- Page 18: “recently developed” is double This has been corrected

- Page 19: The statement “…current efforts to develop inhibitors are focussed solely on PLpro…” is hard to verify or falsify and should be rephrased (e.g. “so far published studies have focused on…”).

We have rephrased this sentence

- Page 23: 2YT media? Changed

- Page 25: Under “Qualitative protease assay”: Something is wrong in the first sentence Corrected

- Centrifugation speed should be given in xg not rpm Changed

- Some of the figures would benefit from aligning panels, adjusting fonts size and style, etc.

We have made changes to the figures to correct these
